# Health professions digital education on clinical practice guidelines: a systematic review by Digital Health Education collaboration

**DOI:** 10.1186/s12916-019-1370-1

**Published:** 2019-07-18

**Authors:** Lorainne Tudor Car, Aijia Soong, Bhone Myint Kyaw, Kee Leng Chua, Naomi Low-Beer, Azeem Majeed

**Affiliations:** 1 Family Medicine and Primary Care, Lee Kong Chian School of Medicine, Nanyang Technological University Singapore, 11 Mandalay Road, Level 18, Clinical Science Building, Singapore, 308232 Singapore; 2 Department of Primary Care and Public Health, School of Public Health, Imperial College London, London, UK; 3 Lee Kong Chian School of Medicine, Nanyang Technological University Singapore, Singapore, Singapore; 4 Medical Education Research Unit, Lee Kong Chian School of Medicine, Nanyang Technological University Singapore, Singapore, Singapore

**Keywords:** Clinical practice guidelines, Health professions education, Systematic review, Digital education

## Abstract

**Background:**

Clinical practice guidelines are an important source of information, designed to help clinicians integrate research evidence into their clinical practice. Digital education is increasingly used for clinical practice guideline dissemination and adoption. Our aim was to evaluate the effectiveness of digital education in improving the adoption of clinical practice guidelines.

**Methods:**

We performed a systematic review and searched seven electronic databases from January 1990 to September 2018. Two reviewers independently screened studies, extracted data and assessed risk of bias. We included studies in any language evaluating the effectiveness of digital education on clinical practice guidelines compared to other forms of education or no intervention in healthcare professionals. We used the Grading of Recommendations, Assessment, Development and Evaluations (GRADE) approach to assess the quality of the body of evidence.

**Results:**

Seventeen trials involving 2382 participants were included. The included studies were diverse with a largely unclear or high risk of bias. They mostly focused on physicians, evaluated computer-based interventions with limited interactivity and measured participants’ knowledge and behaviour. With regard to knowledge, studies comparing the effect of digital education with no intervention showed a moderate, statistically significant difference in favour of digital education intervention (SMD = 0.85, 95% CI 0.16, 1.54; *I*^2^ = 83%, *n* = 3, moderate quality of evidence). Studies comparing the effect of digital education with traditional learning on knowledge showed a small, statistically non-significant difference in favour of digital education (SMD = 0.23, 95% CI − 0.12, 0.59; *I*^2^ = 34%, *n* = 3, moderate quality of evidence). Three studies measured participants’ skills and reported mixed results. Of four studies measuring satisfaction, three studies favoured digital education over traditional learning. Of nine studies evaluating healthcare professionals’ behaviour change, only one study comparing email-delivered, spaced education intervention to no intervention reported improvement in the intervention group. Of three studies reporting patient outcomes, only one study comparing email-delivered, spaced education games to non-interactive online resources reported modest improvement in the intervention group. The quality of evidence for outcomes other than knowledge was mostly judged as low due to risk of bias, imprecision and/or inconsistency.

**Conclusions:**

Health professions digital education on clinical practice guidelines is at least as effective as traditional learning and more effective than no intervention in terms of knowledge. Most studies report little or no difference in healthcare professionals’ behaviours and patient outcomes. The only intervention shown to improve healthcare professionals’ behaviour and modestly patient outcomes was email-delivered, spaced education. Future research should evaluate interactive, simulation-based and spaced forms of digital education and report on outcomes such as skills, behaviour, patient outcomes and cost.

**Electronic supplementary material:**

The online version of this article (10.1186/s12916-019-1370-1) contains supplementary material, which is available to authorized users.

## Introduction

The translation of new research evidence into clinical practice can take up to 17 years [1]. A commonly used strategy that aims to bridge this divide is clinical practice guidelines [2]. Guidelines are evidence synthesis-based recommendations developed to support beneficial clinical practices, reduce unwanted variations and improve patient care outcomes [3, 4]. They are an important source of information for clinicians, designed to help them assimilate, evaluate and adopt evidence into their clinical practice [5]. However, their uptake is still low and dependent on a range of factors relating to the guideline itself (i.e. its complexity, applicability and clarity), healthcare professionals, patients and healthcare organisation [6–8]. Correspondingly, multifaceted interventions targeting these various factors have been shown to be most effective in promoting guideline uptake. While health professions education is an essential part of these multifaceted interventions, it is also still commonly employed as the only guideline dissemination strategy [9]. Evidence to date has mostly focused on traditional learning. Traditional learning for clinical practice guideline adoption was shown to lead to small improvement in desired clinical practices, with more interactive and engaging interventions showing greater effectiveness [10, 11].

Traditional learning, especially the face-to-face type, can be time-consuming, costly and inaccessible [9, 12]. Printed, self-learning resources on the other hand are easily overlooked by busy healthcare professionals [13]. With the relentless growth in research evidence and healthcare complexity, traditional education seems unsustainable in the context of guideline dissemination and training. Digital education, increasingly employed in continuing medical education and professional development, may offer a more flexible, affordable and accessible alternative to traditional learning as it transcends geographical and time constraints. The use of diverse media and delivery devices allows for engaging and interactive learning resources which can be easily updated in line with the new evidence and customised to the individual healthcare professional’s learning needs [14, 15]. By freeing up educators’ and healthcare professionals’ time, digital education may prove to be more cost-effective compared to traditional learning [16, 17]. Past reviews on guideline dissemination and adoption evaluated the effectiveness of traditional education, decision support systems, multifaceted intervention or all digital interventions [9, 11, 18–22]. While digital education is increasingly used in continuing professional development, it is unclear how effective it is in promoting guideline adoption. To address this gap, we decided to undertake a systematic review to investigate the effectiveness and economic impact of digital education for guideline adoption among healthcare professionals.

## Methods

We followed Cochrane recommendations for the conduct of systematic reviews and reported according to the PRISMA guidance [23, 24].

### Study selection

We included RCTs and cluster RCTs that compared digital education to usual education or other forms of digital education to train pre- or post-registration healthcare professionals on clinical practice guidelines. We included healthcare professionals with qualifications found in the Health Field of Education and Training (091) of the International Standard Classification of Education (ISCED-F). We excluded studies of students and/or practitioners of traditional, alternative and complementary medicine. Digital education interventions could be delivered as the only mode of the education intervention or blended with traditional learning (i.e. blended learning). We included offline and online computer-based digital education, digital game-based learning (DGBL), massive open online courses (MOOCs), virtual reality environments (VRE), virtual patient simulations (VPS) and mobile learning (mLearning) [25]. In studies comparing diverse forms of digital education, we differentiated the interventions based on the level interactivity. Interventions with greater control over the learning environment were considered more interactive. We excluded studies on psychomotor skills trainers (PST) as this form of digital education may not be readily available to healthcare professionals. We also excluded studies on interventions that lacked explicit reference to a clinical practice guideline, had an optional digital education component and focused on digital tools for patient management or on computerised decision support systems. Computerised decision support systems are a type of software providing clinicians with decision support in the form of evidence-based, patient-specific recommendations at the point of care [26]. We excluded studies on computerised decision support systems as they have a different underlying principle compared to digital education by being available at the point of care, providing patient-specific recommendations, being integrated with patient data etc. No restrictions on outcomes were applied.

We extracted data on the following primary outcomes:

- Learners’ knowledge, post-intervention. Knowledge is defined as learners’ factual or conceptual understanding measured using change between pre- and post-test scores.
- Learners’ skills post-intervention. Skills are defined as learners’ ability to demonstrate a procedure or technique in an educational setting.
- Learners’ attitudes post-intervention towards new competencies, clinical practice or patients (e.g. recognition of moral and ethical responsibilities towards patients). Attitude is defined as the tendency to respond positively or negatively towards the intervention.
- Learners’ satisfaction post-intervention with the learning intervention (e.g. retention rates, dropout rates, survey satisfaction scores). This can be defined as the level of approval when comparing the perceived performance of digital education compared with one’s expectations.
- Change in healthcare professional’s practice or behaviour.

We also extracted data on the following secondary outcomes:

- Cost and cost-effectiveness of the intervention
- Patient-related outcomes (e.g. heaviness of smoking index, number of smoking cessation)
- Adverse/unintended effects of the intervention

### Data sources, collection, analysis and risk of bias assessment

This review is part of a global evidence synthesis initiative on digital health professions education for which a wider search strategy was developed (see Additional file 1). The following databases were searched from January 1990 to September 2018: MEDLINE (Ovid), Embase (Ovid), Central Register of Controlled Trials (CENTRAL) (Cochrane Library), PsycINFO (EBSCO), Educational Resource Information Centre (ERIC) (EBSCO), CINAHL (EBSCO) and Web of Science Core Collection (Thomson Reuters). The rationale for using 1990 as the starting year for our search was because preceding this year, the use of the computers was largely restricted to very basic functions. No language or publication restrictions were applied. We searched reference lists of all included studies and relevant systematic reviews. We also searched the International Clinical Trials Registry Platform, Search Portal and Current Controlled Trials metaRegister of Controlled Trials to locate unpublished or ongoing trials. We contacted the relevant investigators for missing information. Search results from different sources were combined in a single library, and duplicate records were removed. Two reviewers individually screened titles and abstracts identified by the searches. Full texts of potentially relevant articles were obtained and assessed for inclusion independently by two reviewers. Where data was missing or incomplete, reviewers were contacted for additional information. Any disagreements were settled through discussion between the two reviewers with a third reviewer acting as an arbiter.

Two reviewers extracted the data independently using a standardised data extraction form which was piloted and amended based on feedback. Data was extracted on study design, participants’ demographics, type of digital education, intervention content and outcomes. We contacted study authors in the event of any ambiguous or missing information. Disagreements between reviewers were resolved by discussion. A third reviewer acted as an arbiter in cases where disagreements persisted.

The methodological quality of included RCTs was independently assessed by two reviewers using the Cochrane Risk of Bias Tool which includes the following domains: (1) random sequence generation, (2) allocation concealment, (3) blinding of participants to the intervention, (4) blinding of outcome assessment, (5) attrition, (6) selective reporting and (7) other sources of bias (i.e. baseline imbalances) [23]. The following five additional criteria were included for the assessment of cluster RCTs: (1) recruitment bias which can occur when individuals are recruited to the trial after the clusters have been randomised, (2) baseline imbalance, (3) loss of clusters, (4) incorrect analysis and (5) comparability with individually randomised trials to make sure intervention effects are not overestimated due to ‘Herd effect’ or any such reasons as recommended by the Cochrane Handbook for Systematic Reviews of Interventions [23].

### Data synthesis and analysis

We included post-intervention outcome data in our review for the sake of consistency as this is the most commonly reported form of findings in the included studies. We also reported separately the change score data from the included studies. For continuous outcomes, we reported the standardised mean differences (SMDs) and associated 95% CIs across studies. Standardised mean difference was used as a summary statistic as the outcomes in the included studies were measured differently. We were unable to identify a clinically meaningful effect size from the literature specifically for digital education interventions. Therefore, in line with other evidence syntheses of educational research, we interpreted SMDs using Cohen’s rule of thumb: < 0.2 no effect, 0.2–0.5 small effect size, 0.5–0.8 medium effect size and > 0.80 large effect size [23, 27, 28]. For dichotomous outcomes, we summarised relative risks and associated 95% CIs across studies. Subgroup analyses were not feasible due to the limited number of studies within respective comparisons, and outcomes. We employed the random-effects model in our meta-analysis. The *I*^2^ statistic was employed to evaluate heterogeneity, with *I*^2^ < 25%, 25–75% and > 75% to represent low, moderate and high degree of inconsistency, respectively [23]. The meta-analysis was performed using Review Manager 5.3 (Cochrane Library Software, Oxford, UK) [23]. We reported the findings in line with the PRISMA reporting standards [24]. We assessed and reported the quality of the evidence for each outcome, using the following GRADE assessment criteria: risk of bias, inconsistency, imprecision, indirectness and publication bias. Two authors independently assessed the quality of the evidence. We rated the quality of the body of evidence for each outcome as ‘high’, ‘moderate’ and ‘low’. We prepared ‘Summary of findings’ tables for each comparison to present the findings and the quality of the evidence (Additional file 1) [29]. We were unable to pool the data statistically using meta-analysis for some outcomes (e.g. skills, behaviour) due to high heterogeneity in types of participants, interventions, comparisons, outcomes, outcome measures and outcomes measurement instruments. We presented those findings in the form of a narrative synthesis. We organised the studies by the comparisons and outcomes. We transformed the data expressed in different ways into a common statistical format. We tabulated the results to identify patterns in data across the included studies focusing on both the direction as well as the effect size where possible. In addition, we displayed all the available behaviour change outcome data in a forest plot without a meta-analysis as a visual summary (see Additional file 1). In some studies, behaviour was measured in the same study participants using different approaches and tools. Instead of selecting one outcome or producing a single estimate per study, we present all behaviour change outcome data from the included studies as it focuses on different aspects of clinicians’ behaviour and practice [23].

## Results

**Fig. 1:**
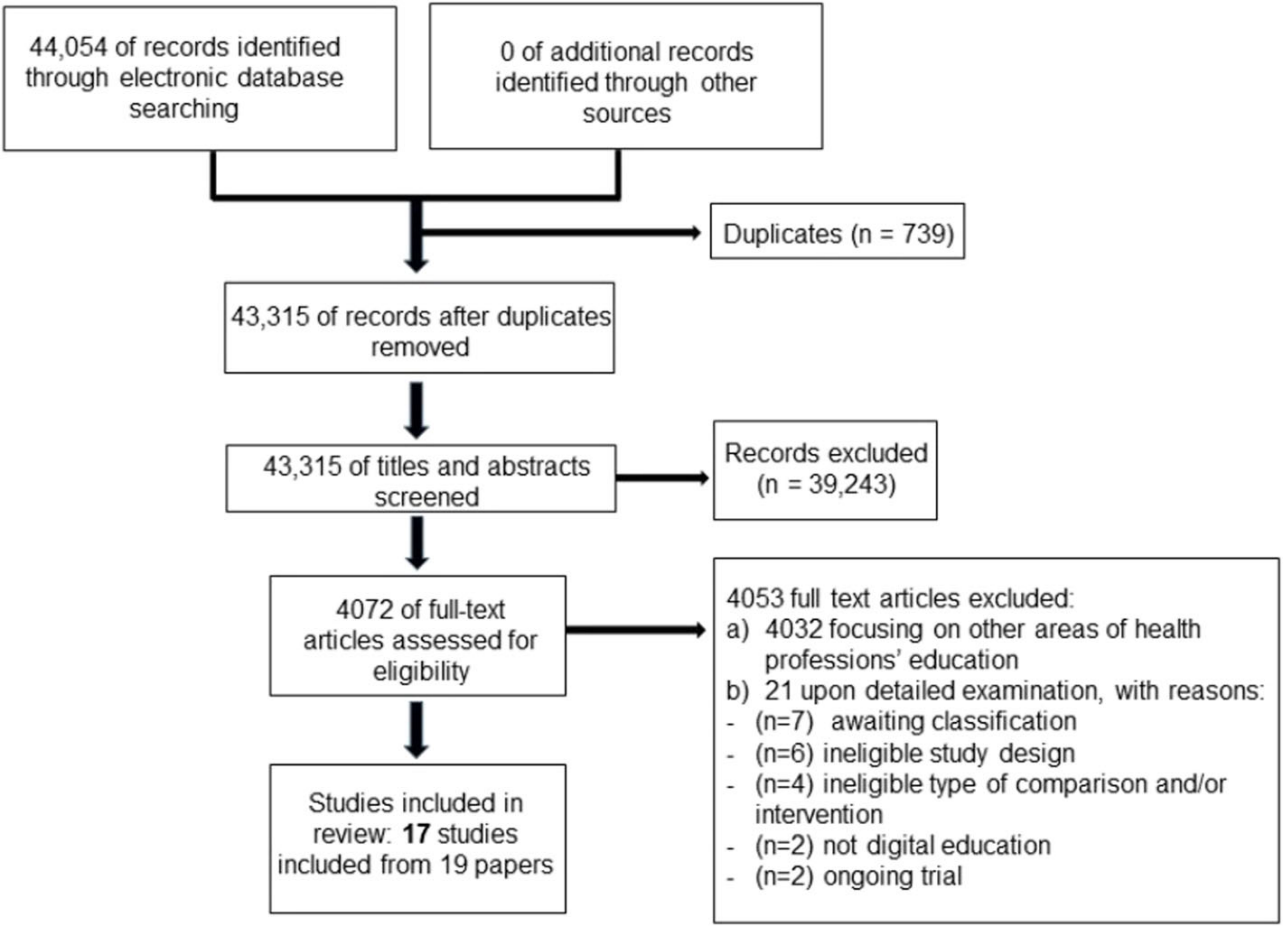
Study flow diagram

Our searches identified a total of 44,054 citations. After screening titles and abstracts, we retrieved full text for 4072 studies focusing on different digital education interventions for health professions education. We identified 40 potentially eligible studies of which 21 studies were excluded for not meeting our inclusion criteria. Seventeen studies from 19 reports, comprising of 14 individually randomised studies and three cluster randomised studies with 2382 participants, were included (Fig. 1, Table 1) [16, 30–43, 46, 47]. One of the included cluster RCTs had three different reports [43]. All seventeen included studies were published in English. Three studies focused on students (nursing students, medical students and emergency medicine students) while the remaining studies targeted post-registration healthcare professionals, mostly primary care physicians [30, 41, 47]. Except for one study from an upper middle-income country [36], all studies were from high-income countries with ten studies from the USA. Sample size ranged from 10 to 1054, with one third of studies having less than 50 participants. Ten studies reported that the intervention was delivered as part of a continuing medical education programme [16, 31, 33–36, 40, 42, 43, 46].

**Table 1:** Characteristics of included studies

Study, design, country	Learning modality	Guideline topic area	No. (type) of participants	Knowledge	Skill	Satisfaction	Behaviour	Patient outcome	Conclusion
Digital education vs no intervention									
Attin et al. [30], RCT, USA	I: Online scenario-based simulation module discussion C: No intervention	Resuscitation	31 nursing students	–	Post-test mean score on a performance-based test SMD 0.93 [0.18, 1.68]	–	–	**–**	Skills: digital education > no intervention
Butzlaff et al. [31], RCT, Germany	I: Online/offline learning module C: No intervention	Dementia, congestive heart failure, urinary tract infection and prevention of colorectal carcinoma	72 primary care physicians	Post-test mean score on a 25-item MCQ SMD 0.43 [− 0.03, 0.90]	–	–	–	**–**	Knowledge: digital education = no intervention
Kerfoot et al. [32], RCT, USA	I: Email-delivered, spaced education C: No intervention	Prostate cancer screening	95 primary care clinicians (physicians, nurse practitioners, physician assistants)	Post-test mean score on a 19-item MCQ SMD 1.52 [1.06, 1.97]	–	–	Proportion of inappropriate PSA screening based on a per patient record RR 0.75 [0.69, 0.82]	–	Knowledge: digital education > no intervention Behaviour: digital education > no intervention
Stewart et al. [33], RCT, USA	I: Online module and discussion C: No intervention	Preventive health practices for peri-menopausal patient, diabetic care	58 primary care physicians	Post-test mean score on a 21-item questionnaire for preventive health practices for peri-menopausal women SMD 0.84 [0.30, 1.38] Post-test mean score on a 22-item questionnaire for type 2 diabetic care SMD 0.32 [− 0.30, 0.84]	–	–	Post-test mean scores from Chart audit for quality of practice on prevention for peri-menopausal patient SMD 0.35 [− 0.18, 0.87] Post-test mean scores from Chart audit for quality of practice on diabetes care SMD 0.01 [− 0.51, 0.52] Post-test mean scores assessed by undercover standardised patients for physician behaviours for prevention case SMD 0.25 [− 0.27, 0.77] Post-test mean scores assessed by undercover standardised patients for physician behaviours for diabetes case SMD 0.23 [− 0.28, 0.75]	–	Knowledge: digital education ≥ no intervention Behaviour: digital education = no intervention
Digital education vs traditional learning									
Bell et al. [34], RCT, USA	I: Online module C: Printed resources (including guidelines)	Care after myocardial infarction	162 primary care and internal medicine residents	Post-test mean score on MCQ SMD 0.22 [− 0.08, 0.53]	–	Post-test mean score from a 5-item survey SMD 1.41 [1.06, 1.75]	–	–	Knowledge: digital education = traditional learning Satisfaction: digital education > traditional learning
Fordis et al. [35], RCT, USA	I: Online module C: Small-group tutorial	Cholesterol management in adults	103 primary care physicians	Post-test mean estimates on a 39-item MCQ and fixed choice responses	–	Proportion of participants rating the learning experience as “(very) good” RR 0.93 [0.85, 1.02]	Proportion of patients screened for dyslipidaemia as per patient record RR 1.02 [0.95, 1.10] Proportion of patients treated for dyslipidaemia as per patient record RR 1.05 [0.94, 1.16]*	–	Knowledge: digital education = traditional learning Behaviour: digital education = traditional learning Satisfaction: digital education = traditional learning
Hemmati et al. [36], RCT, Iran	I: PowerPoint presentation C: Lecture	Resuscitation	80 physicians	Post-test mean score on a 20-item MCQ SMD 0.43 [− 0.02, 0.87]	–	Post-test mean score from a 15-item survey SMD 3.48 [2.77, 4.18]	–	–	Knowledge: digital education = traditional learning Satisfaction: digital education > traditional learning
Jousimaa et al. [37], cRCT, Finland	I: CD-ROM-based guidelines C: Printed guidelines	Consultation practice behaviours	139 physicians	–	–	–	Proportion of laboratory examinations compliant with guidelines from a computer file log RR 1.01 [0.98, 1.03] Proportion of radiological examinations compliant with guidelines from computer file log RR 1.01 [0.99, 1.02] Proportion of physical examinations compliant with guidelines from computer file log RR 0.98 [0.96, 1.00] Proportion of other examinations compliant with guidelines from computer file log RR 0.93 [0.85, 1.01] Proportion of procedures compliant with guidelines from computer file log RR 0.95 [0.85, 1.05] Proportion of Physiotherapy sessions compliant with guidelines from computer file log RR 0.98 [0.85, 1.12] Proportion of non-pharmacologic treatment compliant with guidelines from computer file log RR 0.96 [0.87, 1.06] Proportion of pharmacological treatments compliant with guidelines from computer file log RR 0.98 [0.95, 1.01] Proportion of referrals compliant with guidelines from computer file log RR 1.01 [0.99, 1.02]	–	Behaviour: digital education = traditional learning
Nurse [38], RCT, USA	I: PowerPoint presentation C: Lecture	Pushing and positioning during labour	10 nurses	Post-test mean score on a survey SMD − 0.79 [− 2.11, 0.53]	–	Post-test mean score on Allen’s semantic differential scale SMD 2.26 [0.49, 4.04]	–	–	Knowledge: digital education = traditional learning Satisfaction: digital education > traditional learning
Schwid et al. [39], RCT, USA	I: Online simulation module C: Printed guidelines	Resuscitation	45 anaesthesiology residents	–	Post-test mean score on Megacode performance checklist SMD 1.13 [0.50, 1.76]	–	–	–	Skills: digital education > traditional learning
Shenoy [40], cRCT, USA	I: Online modules C: Printed guidelines	Physical therapy	45 physiotherapists	Mean change score on a 38-item MCQ MD 0.04 [−1.22, 1.31]	–	–	Proportion of patients who received all interventions per guideline as per patient records RR 0.84 [0.45, 1.60] Proportion of patients who received most intervention per guideline as per patient records RR 1.01 [0.75, 1.37] Proportion of patients who received at least one intervention per guideline as per patient records RR 1.35 [0.47, 3.87]	Post-test mean score Patient Quality of life from Oswestry Disability Questionnaire MD − 1.82, [− 7.37, 3.72]	Knowledge: digital education = traditional learning Behaviour: digital education = traditional learning Patient outcomes: digital education = traditional learning
Stephan et al. [41], RCT, Germany	I: Video demonstration C: Peer teaching	Paediatric basic life support	88 medical students	–	Post-test mean score on OSCE examination SMD − 3.72 [− 4.42, − 3.02]	–	–	–	Skills: digital education < traditional learning
Digital education (more interactive) vs digital education									
Bonevski et al. [42], RCT, Australia	I: Online module with performance feedback C: Online module	Screening behaviour for cholesterol, blood pressure and cervical screening and identification of risk behaviours (smoking, alcohol consumption, benzodiazepine use)	19 primary care physicians	–	–	–	Proportion of patients with cholesterol screening as per physician checklist RR 1.35 [1.12, 1.63] Proportion of patients accurately identified for alcohol consumption as per physician checklist RR 1.14 [1.01, 1.28] Proportion of patients with BP screening as per physician checklist RR 1.08 [0.99, 1.18] Proportion of patients accurately identified for long-term benzodiazepine use as per physician checklist RR 1.14 [0.91, 1.43] Proportion of patients accurately identified for smoking as per physician checklist RR 1.01 [0.87, 1.19] Proportion of patients accurately identified for benzodiazepine use as per physician checklist RR 1.03 [0.93, 1.15] Proportion of patients with BP screening as per physician checklist RR 1.06 [0.87, 1.30]	–	Behaviour: digital education (more interactive) ≥ digital education
Billue et al. [43], cRCT, USA Crenshaw et al. [44], cRCT, USA Estrada [45], cRCT, USA	I: Website with performance feedback and reminders C: Online resources	Glucose, blood pressure and lipids control	205 primary care physicians	–	–	–	Rate of medication intensification for HbA1C control as per patient records RR 1.05 [0.88, 1.24] Rate of medication intensification for BP control as per patient records RR 0.98 [0.75, 1.27] Rate of medication intensification for LDL control as per patient records RR 1.35 [0.94, 1.93]	Proportion of patients with optimal control of HbA1C (< 7%) as per patient records RR 1.10 [0.98, 1.24] Proportion of patients with optimal control of BP as per patient records RR 1.10 [0.92, 1.32] Proportion of patients with optimal control of LDL as per patient records RR 0.95 [0.86, 1.05]	Behaviour: digital education (more interactive) = digital education Patient outcome: digital education (more interactive) = digital education
Kerfoot et al. [46], RCT, USA	I: Email-delivered, spaced education game C: Online resources	Blood pressure control	111 primary care physicians	Post-test mean score on a 24-item MCQ SMD 0.81 [0.43, 1.20]	–	–	Rate of monthly medication intensification as per patient records RR 0.95 [0.54, 1.67] Rate of monthly lifestyle counselling as per patient records RR 0.91 [0.66, 1.26]	Duration of the hypertensive period as per as per patient records HR 1.043 [1.007, 1.081]	Knowledge: digital education (more interactive) > digital education Behaviour: digital education (more interactive) = digital education Patient outcome: digital education (more interactive) > digital education
Leszczynski et al. [47], RCT, Poland	a) Online modules with audio-visual material, a questionnaire and feedback b) Online modules with audio-visual material c) Online modules with textual-graphical material	Resuscitation	65 emergency medicine students	Post-test mean change score on a 30-item MCQ (a vs c) SMD 0 [− 0.60, 0.60] Post-test mean change score on a 30-item MCQ (a vs b) SMD 0.10 [− 0.5, 0.70] Post-test mean change score on a 30-item MCQ (b vs c) SMD −0.10 [− 0.72, 0.52]	–	–	–	–	Knowledge: digital education (more interactive) = digital education
Schroter et al. [16], RCT, UK	I: Online module and knowledge-based feedback and customization C: Online resources	Diabetes management	1054 physicians and nurses	Post-test mean score on a 19-item MCQ SMD − 0.09 [− 0.21, 0.03]	–	–	Self-reported practice change via survey RR 0.93 [0.83, 1.04]		Knowledge: digital education (more interactive) = digital education Behaviour: digital education (more interactive) = digital education

*Abbreviations*: *BP* blood pressure, *cRCT* cluster randomised controlled trial, *HR* hazard ratio, *LDL* low-density lipoproteins, *MCQ* multiple choice questionnaire, *RCT* randomised controlled trial, *RR* risk ratio, *SMD* standardised mean difference, *vs* versus

Eight studies compared digital education to traditional learning (i.e. lectures, paper-based self-study materials and small-group tutorial) [34–41], four studies compared digital education digital education to no intervention [30–33] and five studies compared more to less interactive forms of digital education [16, 42, 43, 46, 47]. Digital interventions mostly consisted of educational materials with low or no interactivity. Nine studies focused on online modules (i.e. sequenced collection of subject-related, multimedia materials) with or without feedback and discussions [16, 31, 33–35, 40, 42, 43, 47]; two studies each on online simulation [30, 39], spaced education in the form of regular email-delivered surveys [32, 46] and PowerPoint presentations [36, 38]; and one study each on computer-based offline video [41] and CD-ROM-delivered intervention [37]. The educational content in the included studies spanned resuscitation, paediatric basic life support, diabetes, preventive care, labour management, myocardial infarction management, hypertension management, physical therapy, prostate cancer screening, dementia, urinary tract infection and heart failure. Four studies focused on more than one guideline, i.e. two, four, five and 1100 guidelines [31, 33, 37, 42]. Included studies measured knowledge, skills, satisfaction, behaviour and patient outcomes. None of the studies reported attitudes, cost-related outcomes or adverse/unintended effects. All studies measured outcomes immediately after the intervention. In addition, six studies measured long-term knowledge retention ranging from 1 to 9 months post-intervention [32–35, 38, 47] and two studies measured long-term behaviour change [32, 33].

**Fig. 2:**
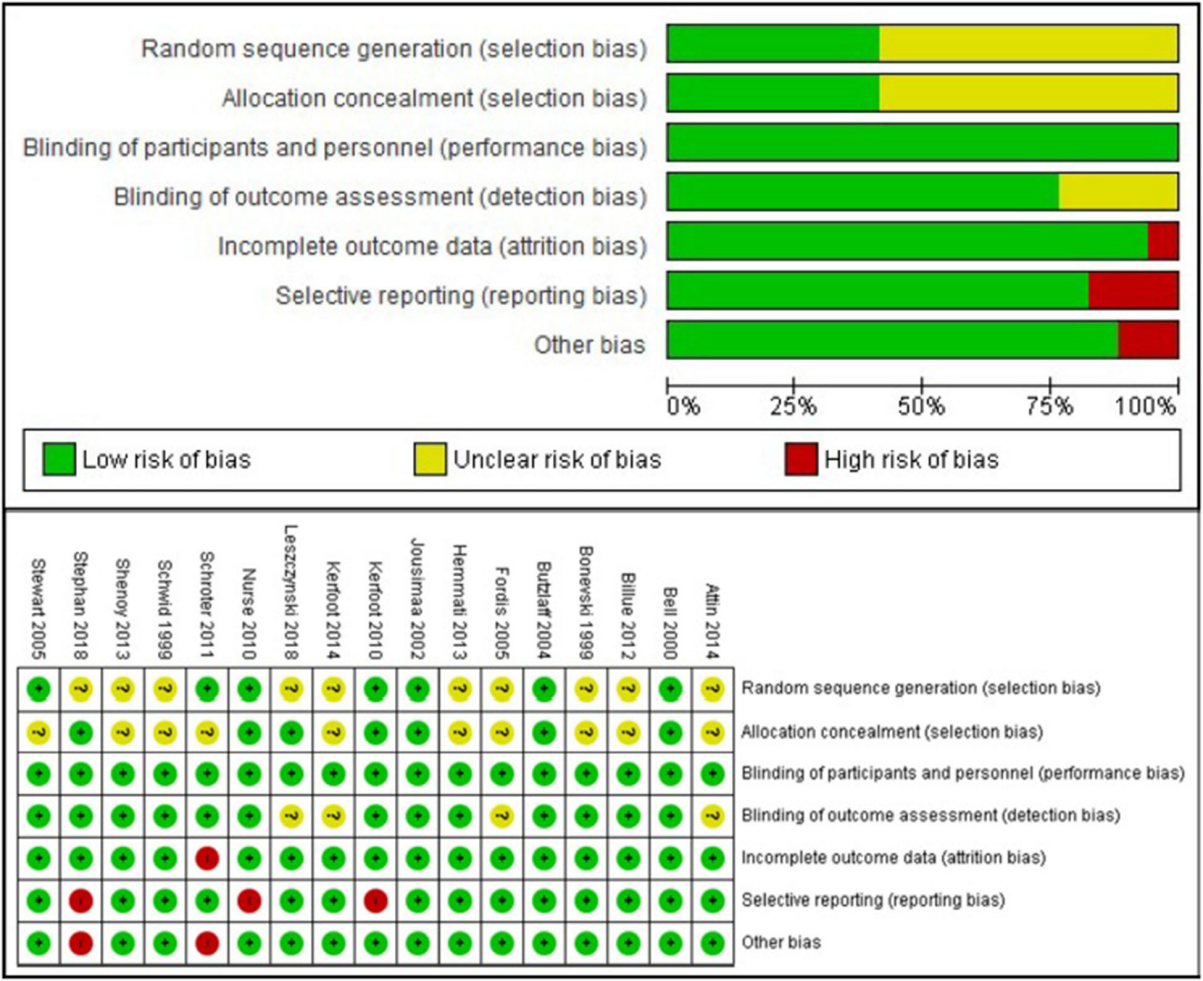
Risk of bias summary

Half of the studies had unclear or high risk of bias for random sequence generation and more than half had unclear risk for allocation concealment due to missing information, resulting in general unclear risk of bias for selection bias (see Fig. 2). Outcome assessment was mostly done by non-blinded assessors and without the use of validated instruments. For cluster RCTs, the risk of bias was overall low. The quality of evidence ranged from low to moderate and was downgraded because of risk of bias, imprecision and/or inconsistency (see Additional file 1).

### Digital education vs no intervention

**Fig. 3:**
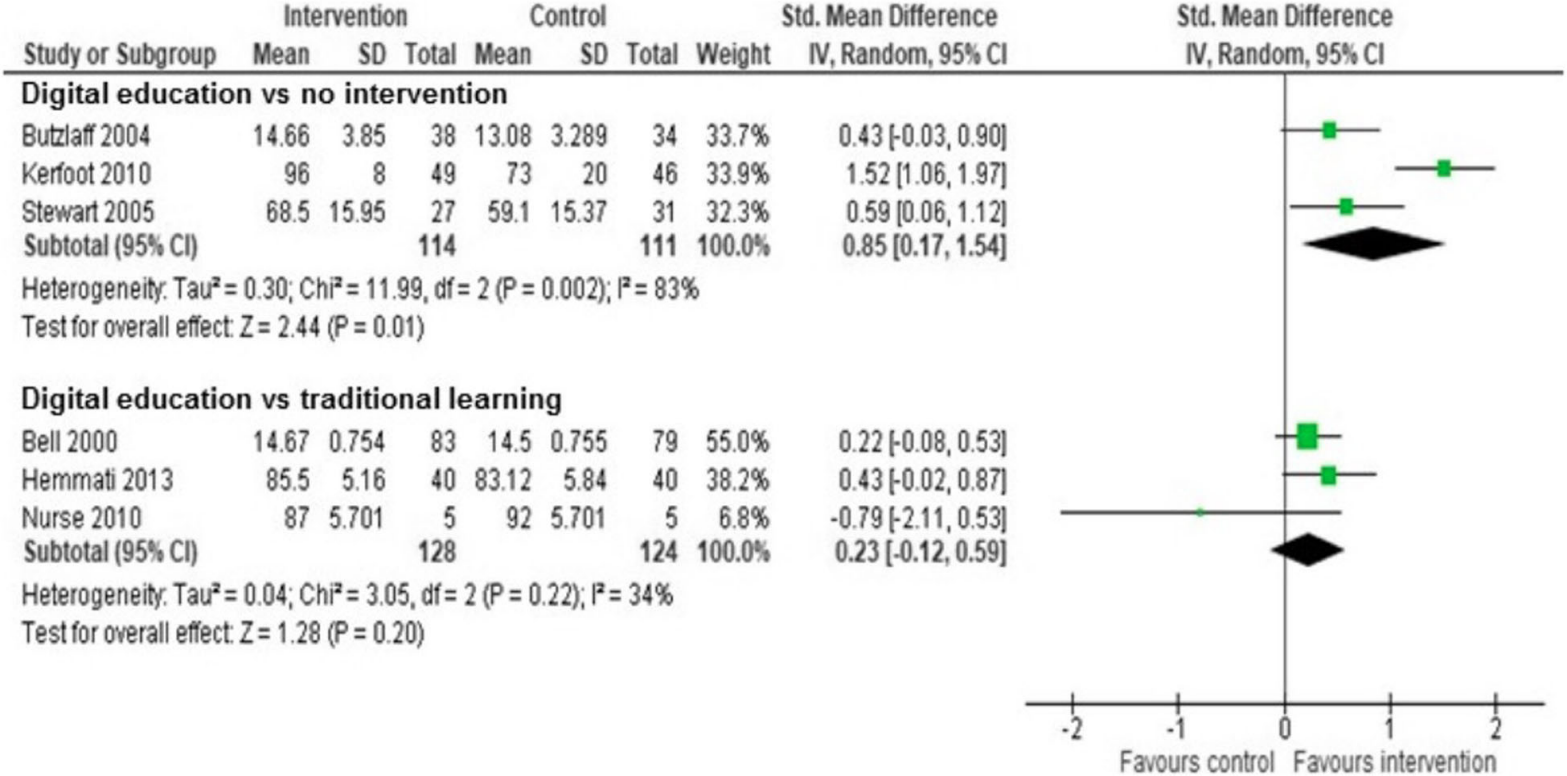
Forest plot of knowledge outcome comparing digital education on clinical practice guidelines to no intervention or traditional learning

Four studies compared the effects of digital education for clinical practice guideline adoption to no intervention (Table 1). Three of these four studies evaluated participants’ knowledge [31–33]. The pooled analysis of these studies showed large beneficial effect of digital education interventions for clinical practice guideline adoption on knowledge scores (SMD = 0.85, 95% CI 0.16, 1.54; *I*^2^ = 83%, moderate quality of evidence) (Fig. 3). The high observed heterogeneity was largely driven by a study on spaced education via emails showing large improvement in the intervention group (SMD = 1.52, 95% CI 1.06, 1.97) [32] and CIs that poorly overlap with the CIs from the other two studies in this analysis. The two remaining studies that evaluated online modules and case-based discussion reported mixed results [31, 33]. One study measuring long-term knowledge retention at 6 months post-intervention [33] reported moderate beneficial effect of the digital education intervention group when compared to no intervention (SMD = 0.73, 95% CI 0.09, 1.38).

Only one study (*n* = 31), evaluating the use of a simulation-based module, measured participants’ skills post-intervention and reported a large beneficial effect of digital education (SMD = 0.93, 95% CI 0.18–1.68, low quality of evidence) [30]. The effect of digital education on healthcare professionals’ behaviour was reported in two studies with mixed findings [32, 33]. Study on the use of spaced education via emails reported improvement in healthcare professional’s behaviour (RR = 0.75, 95% CI 0.69, 0.828) [32]. Conversely, the study on the use of online module and discussions reported no difference in healthcare professionals’ behaviour [33]. The same two studies also reported long-term data for behavioural change outcome. The follow-up behavioural change findings in these studies were consistent with those immediately post-intervention with one study evaluating an online module reporting no difference between the groups at 6 months [33], and the other study on spaced education still favouring the intervention group at 18 months post-intervention [32].

None of the studies reported on attitudes, adverse effect, patient outcomes or cost outcomes.

### Digital education vs traditional learning

Eight studies compared the effects of digital education for clinical practice guideline adoption to traditional learning (Table 1) [34–41]. Five of these eight studies (*n* = 405) measured knowledge [34–36, 38, 40]. The pooled estimate from three studies reporting post-intervention data showed a small statistically non-significant effect on knowledge scores in the digital education group compared to traditional learning (SMD = 0.23, 95% CI − 0.12, 0.59; *I*^2^ = 34%, moderate quality of evidence) (Fig. 3). The moderate heterogeneity was due to a small, pilot study with very imprecise findings [38] as shown by its wide CIs that poorly overlap with the CIs from the other two studies in this analysis. The remaining two studies without post-intervention data also reported no difference between the groups immediately post-intervention although one of them reported that the intervention group scored slightly higher than the control group when averaged across baseline, post-intervention and follow-up measurement [35]. Three studies also measured long-term knowledge retention 1 to 6 months post-intervention and reported no difference between the groups in two studies [35, 38] and moderate improvement in the digital education group in one study [34].

Of four studies evaluating participants’ satisfaction with the intervention [34–36, 38], three studies reported large beneficial effect of digital education compared to a lecture or printed resources [34, 36, 38]. One study, employing interactive small-group learning as a control, reported no difference [35].

Two studies (*n* = 133) reported post-intervention skills outcome [39, 41]. One study (*n* = 45) evaluating the use of simulation-based learning module reported large beneficial effect of digital education (SMD = 1.13, 95% CI 0.50, 1.76, moderate quality of evidence) in comparison to printed guidelines [39]. The other study assessed the effectiveness of computer-based video demonstration compared to peer teaching and reported higher post-intervention skills score in the control group (SMD = − 3.72, 95% CI − 4.42, 3.02, low quality of evidence) [41]. Three studies analysed the healthcare professionals’ behaviour change and reported no difference between the groups (Additional file 1) [35, 37, 40]. One study assessed patient outcomes and reported no differences between groups [40]. None of the included studies reported on attitudes, adverse effects or cost outcomes.

### Digital education (more interactive) vs digital education (less interactive)

Five studies compared different configurations of digital education interventions (Table 1) [16, 42, 43, 46, 47]. Four studies evaluated online modules with performance-based or knowledge-based feedback [16, 42, 43, 47], and one study evaluated email-delivered, spaced education game [46]. The control interventions were either less interactive form of the digital education or non-interactive, online resources. Four studies measured behaviour and largely reported no difference between the groups (Fig. 4, Table 1) [16, 42, 43, 46]. Of three studies measuring knowledge [16, 46, 47], only one study on spaced education game favoured intervention (SMD = 0.81, 95% CI 0.43–1.20, moderate quality of evidence) [46]. This study also reported a modest improvement in patient outcomes. One study reported knowledge growth rate and reported no difference in mean change scores between the most interactive intervention groups and the less interactive control groups [16]. This study also reported no differences in satisfaction scores between the groups. One study reported moderate improvement in knowledge growth retention at 30-day follow-up in the more interactive form of digital education intervention compared to less interactive one (SMD = 0.63, 95% CI 0.01; 1.24) [47]. The same study reported higher satisfaction in the more interactive group at follow-up. No studies reported attitudes, adverse effect or cost outcomes.

## Discussion

We identified 17 studies evaluating the effectiveness of digital education for clinical practice guideline adoption among healthcare professionals. Studies mostly focused on primary care physicians, computer-based educational interventions with low interactivity and measurement of participants’ knowledge and behaviour. With regard to knowledge, studies comparing the effect of digital education with no intervention showed a moderate, statistically significant difference in favour of digital education. Studies comparing the effect of digital education with traditional learning on knowledge showed a small, statistically non-significant difference in favour of digital education. The digital education group was more satisfied than the traditional learning group. Overall, there was little or no change in healthcare professionals’ behaviour, except in one study favouring the email-delivered, spaced education intervention group. Of three studies measuring patient outcomes, only one study on spaced education game intervention reported a modest improvement in the intervention group. The quality of evidence ranged from low to moderate across outcomes due to risk of bias, inconsistency in the findings and/or imprecision of the outcome data.

The evaluated digital educational interventions had diverse formats. The existing literature on the effectiveness of traditional learning for clinical practice guideline adoption shows that interactive approaches may be more effective than passive guideline dissemination [10, 11]. In our review, most digital education interventions focused on passive dissemination of resources with no or low interactivity, e.g. PowerPoint presentations or computer-based text [31, 34, 36–38]. Three studies compared the effectiveness of more interactive digital education interventions in the form of spaced education via email or online simulation to no intervention or traditional learning and reported large beneficial effect in the intervention group [30, 32, 39]. However, most studies comparing more interactive to less interactive forms of digital education reported no statistically significant difference between the groups. The interactive component of these digital education interventions was mostly in the form of performance feedback. The only study favouring the more interactive form of digital education knowledge and patient outcomes compared spaced education via emails to passive online resources. Based on these findings, future research should explore further the effectiveness of spaced digital education and simulation on clinical practice guidelines compared to other forms of education.

Included studies on interventions with limited interactivity reported various challenges relating to participants’ attrition and limited usage of the resources. Two studies evaluating the use of websites and online modules reported high attrition rates [16, 31]. In a study on a computer-based module with performance feedback, participants considered the intervention too time-demanding [42]. A study evaluating a digital education intervention in the form of a non-interactive, digitally presented clinical practice guidelines reported that more than half of participants accessed the provided resources either once or never [31]. Another study with computer-based resources customised to participants with hyperlinks reported that less than 60% of provided educational material was accessed [34]. Conversely, more interactive interventions reported lower attrition rates and higher participants’ engagement [39, 44].

The effectiveness of digital education for guideline adoption may also depend on the type of content it focuses on. Learners may already have high baseline knowledge on some topics such as diabetes which may lead to a ceiling effect in the knowledge score [33]. In addition, educational interventions focusing on several guidelines concurrently may lead to increased cognitive load and therefore be less effective than those focusing on a single guideline. Yet with the growing prevalence of chronic illnesses, ageing population and multi-morbidity, single guidelines are perceived as impractical and unhelpful [48]. Instead of passive dissemination of several distinct guidelines, digital education offers opportunity for seamless and engaging education and blending of diverse guidelines, using for example scenario-based learning and simulation. Notably, none of the included studies in our review evaluated novel educational modalities enabling simulated learning such as virtual or augmented reality. Also, none of the studies used mobile devices for delivery of digital education interventions. Mobile-delivered education may be more suitable to meet the needs of healthcare professionals by enabling easy, on-the-go access to training. These diverse digital education modalities may help promote better participant engagement and prove a more effective approach to health professions’ training on guidelines and should be evaluated in the future.

Our review has several limitations. Randomised controlled trials included in this review mostly lacked information on randomisation method, allocation concealment or blinding method. Included studies also largely reported post-intervention data, so we could not calculate pre-post intervention change data nor ascertain whether the intervention groups were matched at baseline for key characteristics and outcome measure scores. In studies reporting pre-post intervention change data, we extracted post-intervention data to ensure consistency in the presentation of findings across the studies included in this review. Studies with pre-post intervention change data reported an improvement from the baseline, but the findings were in all cases consistent with the post-intervention data. Furthermore, in our review, we only focused on studies on clinical practice guidelines and may have missed studies that use other forms of evidence-based recommendations or do not explicitly cite a clinical practice guideline. In line with other systematic reviews on digital education interventions, we have encountered substantial heterogeneity in terms of the intervention, participants, outcomes and comparisons [49]. Given such heterogeneity, our findings have to be interpreted with caution. In addition, our review focuses only on digital education on guidelines and its findings are not applicable to other digital interventions such as computerised decision support systems. Finally, some studies reported that digital education interventions were delivered as part of a continuing medical education programme which may have affected their findings. Our review strengths include a comprehensive and sensitive search as well as clear inclusion and exclusion criteria encompassing a broad range of participants, outcomes and interventions. We also performed parallel, independent and reproducible screening, data extraction and rigorous risk of bias assessment.

## Conclusion

Digital education on clinical practice guidelines seems to be more effective than no intervention and at least as effective as traditional learning in terms of participants’ knowledge. Participants reported higher satisfaction with digital education compared to traditional learning. Yet, digital education overall led to little or no difference in health professionals’ behaviour as compared to control interventions. Higher interactivity and engagement in digital education interventions for clinical practice guideline adoption may lead to larger educational gains. Future research should aim to determine the effectiveness of novel modalities (e.g. mobile learning and virtual reality), as well as spaced and interactive formats of digital education, and focus on outcomes such as skills, attitudes, cost, behaviour and patient outcomes. There is a need for high-quality, well-reported RCTs with a clear presentation of the random sequence generation and allocation concealment approach as well a detailed description of the intervention and the control. Future studies should report pre-post intervention change outcome data, use validated outcome measurement tools and assess learners’ retention as well as long-term outcomes.

## Additional file

Additional file 1: Supplementary information including MEDLINE (Ovid) search strategy, Summary of findings tables and Forest plot of all behavioural change outcomes. (DOCX 155 kb)

## Data Availability

This systematic review included the data extracted from the primary studies. The whole set of data extraction sheet is available upon request.

## Abbreviations

BP: Blood pressure
HR: Hazard ratio
LDL: Low-density lipoproteins
MCQ: Multiple choice questionnaire
RCT: Randomised controlled trial
RR: Risk ratio
SMD: Standardised mean difference
