# Supplementary material for: Health professions digital education on clinical practice guidelines: a systematic review by Digital Health Education collaboration
Source: BMC Med. 2019 Jul 18;17:139. doi: 10.1186/s12916-019-1370-1 (PMC6637541; doi:10.1186/s12916-019-1370-1)

# Supplementary Information

Tudor Car L, Soong A, Kyaw BM et al. Health professions digital education on clinical practice guidelines: a systematic review by Digital Health Education collaboration

### MEDLINE (Ovid) Search Strategy

1. exp education, professional/ not education, veterinary/

2. Education, Predental/

3. Education, Premedical/

4. exp Students, Health Occupations/

5. ((medic* or premedic* or dent* or laborator* or predent* or midwi?e* or nurs* or nutrition* or orthop* or podiat* or pharmac* or psycholog* or psychiatr* or health or healthcare or occupational therap* or physiotherap* or physical therap* or clinical or surg* or radiolog* or obstetric* or gyn?ecolog* or orthodont* or An?esthesi* or Dermatolog* or Oncolog* or Rheumatolog* or Neurolog* or Patholog* or P?ediatric* or Cardiolog* or Urolog*) adj3 (student* or graduate* or undergraduate* or staff or personnel or practitioner* or clerk* or fellow* or internship* or residen* or educat* or train* or novice* or tutor*)).tw,kf.

6. or/1-5

7. Computer-Assisted Instruction/

8. exp Internet/

9. Computer Simulation/

10. Patient Simulation/

11. software/

12. Mobile Applications/

13. User-Computer Interface/

14. Video Games/

15. Web Browser/

16. Education, Distance/

17. Computers/

18. exp Microcomputers/

19. exp Cell Phones/

20. Games, Experimental/

21. exp Models, Anatomic/

22. Audiovisual Aids/

23. Educational Technology/

24. Electronic Mail/

25. exp Telemedicine/

26. Telenursing/

27. Telecommunications/

28. Webcasts/

29. exp Videoconferencing/

30. ((computer* or digital* or hybrid or blended or mixed mode or distance or remote* or electronic or mobile or online* or interactiv* or multimedia or internet or web* or virtual* or game* or gaming or Videogame* or Videogaming) adj3 (classroom* or course* or educat* or instruct* or learn* or lecture* or simulat* or train* or teach* or tutor* or platform*)).tw,kf.

31. (Simulat* adj3 (course* or educat* or instruct* or learn* or train* or teach* or platform* or high-fidelity)).tw,kf.

32. e-learn*.tw,kf.

33. elearn*.tw,kf.

34. m-learn*.tw,kf.

35. mlearn*.tw,kf.

36. smartphone*.tw,kf.

37. smart-phone*.tw,kf.

38. ((mobile or cell) adj2 phone*).tw,kf.

39. iphone*.tw,kf.

40. android*.tw,kf.

41. ipad*.tw,kf.

42. Personal digital assistant*.tw,kf.

43. handheld computer*.tw,kf.

44. Mobile App?.tw,kf.

45. Mobile Application?.tw,kf.

46. webcast*.tw,kf.

47. webinar*.tw,kf.

48. flipped classroom*.tw,kf.

49. Serious game*.tw,kf.

50. Serious gaming.tw,kf.

51. Patient Simulat*.tw,kf.

52. Virtual patient*.tw,kf.

53. ((educat* or instruct* or learn* or simulat* or train* or teach* or interactiv*) adj2 technolog*).tw,kf.

54. Massive Open Online Course?.tw,kf.

55. Mooc?.tw,kf.

56. (Canvas network or Coursera or Coursesites or edx or Futurelearn or iversity or miriada x or moodle or novoed or openlearning or open2study or plato or spoc or udacity or pingpong).tw,kf.

57. or/7-56

58. 6 and 57

59. Education.fs.

60. Education/

61. Teaching/

62. Learning/

63. exp Inservice Training/

64. Curriculum/

65. educat*.tw,kf.

66. learn*.tw,kf.

67. train*.tw,kf.

68. instruct*.tw,kf.

69. teach*.tw,kf.

70. or/59-69

71. Health Personnel/

72. exp Allied Health Personnel/

73. Anatomists/

74. "Coroners and Medical Examiners"/

75. exp Dental Staff/

76. exp Dentists/

77. Health Educators/

78. Infection Control Practitioners/

79. Medical Laboratory Personnel/

80. exp Medical Staff/

81. exp Nurses/

82. exp Nursing Staff/

83. Personnel, Hospital/

84. Pharmacists/

85. exp Physicians/

86. Physician*.tw,kf.

87. Doctor*.tw,kf.

88. Nurs*.tw,kf.

89. Surg*.tw,kf.

90. Health Personnel.tw,kf.

91. healthcare professional*.tw,kf.

92. radiolog*.tw,kf.

93. dentist*.tw,kf.

94. Pharmacist*.tw,kf.

95. Hospital Administrator*.tw,kf.

96. Podiatr*.tw,kf.

97. Psycholog*.tw,kf.

98. Psychiatr*.tw,kf.

99. An?esthesi*.tw,kf.

100. Clinician*.tw,kf.

101. Dermatolog*.tw,kf.

102. General practioner*.tw,kf.

103. Cardiolog*.tw,kf.

104. Oncolog*.tw,kf.

105. Rheumatolog*.tw,kf.

106. Neurolog*.tw,kf.

107. Patholog*.tw,kf.

108. P?ediatric*.tw,kf.

109. Physiotherap*.tw,kf.

110. Physical therap*.tw,kf.

111. Occupational therap*.tw,kf.

112. dieti?ian*.tw,kf.

113. Dietetic*.tw,kf.

114. midwi?e*.tw,kf.

115. nutrition*.tw,kf.

116. orthopti*.tw,kf.

117. obstetric*.tw,kf.

118. gyn?ecolog*.tw,kf.

119. orthodont*.tw,kf.

120. Urolog*.tw,kf.

121. or/71-120

122. Health Occupations/

123. exp Allied Health Occupations/

124. Biomedical Engineering/

125. Chiropractic/

126. exp Dentistry/

127. exp Evidence-Based Practice/

128. exp Medicine/

129. exp Nursing/

130. Dietetics/

131. Optometry/

132. Orthoptics/

133. exp Pharmacology/

134. exp Pharmacy/

135. Podiatry/

136. Psychology, Medical/

137. Serology/

138. Specialization/

139. exp Surgical Procedures, Operative/

140. exp Radiography/

141. or/122-140

142. 121 or 141

143. 57 and 70 and 142

144. Psychomotor Performance/

145. motor skills/

146. ((psychomotor or procedural or technical) adj3 skill*).tw,kf.

147. (psychomotor adj3 performance).tw,kf.

148. or/144-147

149. 6 and 148

150. 58 or 143 or 149

151. limit 150 to yr="1990 -Current"

152. randomized controlled trial.pt.

153. controlled clinical trial.pt.

154. randomized.ti,ab.

155. placebo.ti,ab.

156. drug therapy.fs.

157. randomly.ti,ab.

158. trial.ti,ab.

159. groups.ti,ab.

### Summary of Findings Tables

| **Digital education vs no intervention** | | | | |
| --- | --- | --- | --- | --- |
| **Patient or population:** Pre-registration or post-registration healthcare professionals  **Settings:** University or clinical settings  **Intervention:** Digital education  **Comparison:** No intervention | | | | |
| **Outcomes** | **Illustrative comparative risks (95% CI)** | **No of Participants (studies)** | **Quality of the evidence (GRADE)** | **Comments** |
| **Knowledge** Assessed with MCQ, questionnaire (immediately post-intervention, 4 to 36 weeks post-intervention) | The mean score in the intervention group was 0.85 standard deviations higher (0.16 higher to 1.54 higher) than the mean score in the no intervention group. | 225 participants (3 studies) | ⊕⊕⊕⊝  Moderate quality^1^ | Findings from three studies were meta-analyzed showing a large difference in knowledge scores between the groups in favor of digital education (SMD = 0.85, 95% CI: 0.16, 1.54) (Butzlaff 2004, Kerfoot 2010, Stewart 2005). |
| **Skills** Assessed with performance-based test (immediately post-intervention) | The mean score in the intervention group was 0.93 standard deviations higher (0.18 higher to 1.68 higher) than the mean score in the no intervention group. | 31 participants (1 study) | ⊕⊕⊝⊝  Low quality^2,3^ | One study reported higher skills scores in the intervention group, large effect size (SMD of 0.93, 95% CI: 0.18 - 1.68) (Attin 2014). |
| **Satisfaction** | Not estimable | 0 participants (0 studies) | Not estimable | No study assessed participants' post intervention satisfaction scores in both the intervention and control groups. |
| **Behavioral change** Assessed with patient record, chart audit (immediately post-intervention, six to 18 months post-intervention) | Not estimable | 153 participants (2 studies) | ⊕⊕⊝⊝  Low quality^1,2^ | One study reported improvement in health professionals’ behavior in the intervention group compared to no intervention (Kerfoot 2010). The other study reported no difference in post-intervention behavioral outcome between the intervention and control groups (Stewart 2005). |
| **Patient Outcome** | Not estimable | 0 participants (0 studies) | Not estimable | No study assessed patient outcomes in both the intervention and control groups. |
| **CI:** Confidence interval; **RR:** Risk Ratio, SMD: standardized mean difference | | | | |
| GRADE Working Group grades of evidence **High quality:** Further research is very unlikely to change our confidence in the estimate of effect. **Moderate quality:** Further research is likely to have an important impact on our confidence in the estimate of effect and may change the estimate. **Low quality:** Further research is very likely to have an important impact on our confidence in the estimate of effect and is likely to change the estimate. **Very low quality:** We are very uncertain about the estimate. | | | | |

Footnotes

^1^ Rated down by one level for inconsistency: the heterogeneity is high with large variations in effect and lack of overlap among confidence intervals (CIs).

^2^Rated down by one level for study limitations: the risk of bias was unclear for sequence generation and allocation concealment in majority of the studies.

^3^Rated down by one level for imprecision: number of participants (effective sample size) in the studies is less than the number of patients generated by a conventional sample size calculation for a single adequately powered trial (optimal information size).

| **Digital education vs Traditional learning** | | | | |
| --- | --- | --- | --- | --- |
| **Patient or population:** Pre-registration or post-registration healthcare professionals  **Settings:** University or clinical settings  **Intervention:** Digital education  **Comparison:** Traditional learning (printed resources including guidelines, small-group tutorial, lecture, peer teaching) | | | | |
| **Outcomes** | **Illustrative comparative risks (95% CI)** | **No of Participants (studies)** | **Quality of the evidence (GRADE)** | **Comments** |
| **Knowledge** Assessed with MCQ, fixed choice responses (immediately post-intervention, one to six months post-intervention) | The mean score in the intervention group was 0.23 standard deviations higher (-0.12 lower to 0.59 higher) than the mean score in the traditional learning group. | 400 participants (5 studies) | ⊕⊕⊕⊝  Moderate quality^1^ | Findings from three studies (252 participants) were meta-analyzed showing a small, statistically non-significant difference in knowledge scores between the groups 0.23 in favor of digital education (95% CI: -0.12 to 0.59) (Bell 2000, Hemmati 2013, Nurse 2010). Two studies without post-intervention data also found no difference knowledge scores between the groups (Fordis 2005, Shenoy 2013). |
| **Skills** Assessed with performance checklist, OSCE examination (immediately post-intervention, 10 to 12 weeks post-intervention) | Not estimable | 133 participants (2 studies) | ⊕⊕⊝⊝  Low quality^2,3^ | Two studies reported mixed findings. One study comparing online simulation module to the use of printed guidelines reported higher skills score in the intervention group, large effect size (Schwid 1999).  The other study compared video demonstration to peer teaching and reported higher skills score in the control group, large effect size (Stephan 2018). |
| **Satisfaction** Assessed with survey, Allen's semantic differential scale (immediately post-intervention) | Not estimable | 355 participants (4 studies) | ⊕⊕⊝⊝  Low quality^2,3^ | Three studies reported higher post-intervention satisfaction (large effect size) in intervention group compared to control group (Bell 2000, Hemmati 2013, Nurse 2010), while one study reported no differences between the intervention and control groups (Fordis 2005). |
| **Behavioral change** Assessed with patient record, computer file logs (immediately post-intervention) | Not estimable | 287 participants (3 studies) | ⊕⊕⊕⊝  Moderate quality^3^ | All three studies reported no differences in post-intervention behavioral outcome between the intervention and control groups (Fordis 2005, Jousimaa 2002, Shenoy 2013). |
| **Patient outcomes** Assessed with questionnaire (immediately post-intervention) | Not estimable | 45 participants (1 study) | ⊕⊕⊝⊝  Low quality^1,3^ | One study reported no difference in patient outcomes between the intervention and control groups (Shenoy 2013). |
| * **CI:** Confidence interval; **RR:** Risk Ratio; **NA:** Not Applicable, **SMD:** standardized mean difference | | | | |
| GRADE Working Group grades of evidence **High quality:** Further research is very unlikely to change our confidence in the estimate of effect. **Moderate quality:** Further research is likely to have an important impact on our confidence in the estimate of effect and may change the estimate. **Low quality:** Further research is very likely to have an important impact on our confidence in the estimate of effect and is likely to change the estimate. **Very low quality:** We are very uncertain about the estimate. | | | | |

Footnotes

^1^ Rated down by one level for imprecision: number of participants (effective sample size) in the studies is less than the number of patients generated by a conventional sample size calculation for a single adequately powered trial (optimal information size)
^2^ Rated down by one level for inconsistency: the heterogeneity is high with large variations in effect and lack of overlap among confidence intervals (CIs).
^3^Rated down by one level for study limitations: the risk of bias was unclear for sequence generation and allocation concealment in majority of the studies or there was high risk of bias in some of the included studies.

| **Digital education (more interactive) compared with Digital education** | | | | |
| --- | --- | --- | --- | --- |
| **Patient or population:** Post-registration healthcare professionals  **Settings:** University or clinical settings  **Intervention:** Digital education (more interactive)  **Comparison:** Digital education | | | | |
| **Outcomes** | **Illustrative comparative risks (95% CI)** | **No of Participants (studies)** | **Quality of the evidence (GRADE)** | **Comments** |
| **Knowledge**  Assessed with MCQ (immediately post-intervention, 30 days post-intervention) | Not estimable | 1230 participants (3 studies) | ⊕⊕⊝⊝  Low quality^1,2^ | Two studies comparing online module to online resources reported no difference in knowledge scores (Schroter 2011, Leszcynski 2018). One study comparing email delivered spaced education game to online resources reported higher knowledge scores in the intervention group, large effect size (Kerfoot 2014). |
| **Skills** | Not estimable | 0 participants (0 studies) | Not estimable | No study assessed participants' post intervention skills scores in both the intervention and control groups. |
| **Satisfaction** | Not estimable | 0 participants (0 studies) | Not estimable | No study assessed participants' post intervention satisfaction scores in both the intervention and control groups. |
| **Behavioral change** Assessed with physician checklist, patient records, survey (immediately post-intervention) | Not estimable | 1389 participants (4 studies) | ⊕⊕⊝⊝  Moderate quality^2^ | Three studies reported no differences between the groups (Billue 2012, Kerfoot 2014, Schroter 2011). One study reported little or no difference in post-intervention behavioral outcome between the intervention and control groups (Bonevski 1999). |
| **Patient outcomes** Assessed with patient records (immediately post-intervention) | Not estimable | 316 participants (2 studies) | ⊕⊕⊝⊝  Low quality^1,2^ | One study reported improvement in post-intervention patient outcomes with intervention group compared to control group (Kerfoot 2014). The other study reported no differences in post-intervention patient outcomes between intervention and control group (Billue 2012). |
| **CI:** Confidence interval; **RR:** Risk Ratio; **NA:** Not Applicable; **SMD:** standardized mean difference | | | | |
| GRADE Working Group grades of evidence **High quality:** Further research is very unlikely to change our confidence in the estimate of effect. **Moderate quality:** Further research is likely to have an important impact on our confidence in the estimate of effect and may change the estimate. **Low quality:** Further research is very likely to have an important impact on our confidence in the estimate of effect and is likely to change the estimate. **Very low quality:** We are very uncertain about the estimate. | | | | |

Footnotes

^1^Rated down by one level for inconsistency: the heterogeneity is high with large variations in effect and lack of overlap among confidence intervals (CIs).
^2^Rated down by one level for study limitations: the risk of bias was unclear for sequence generation and allocation concealment in majority of the studies or there was high risk of bias in some of the included studies.

### Forest plot of all behavioral change outcomes reported in included studies (including multiple outcomes measured on the same set of participants) without the pooled estimate. *Kerfoot 2010 is the only study in this forest plot in which lower RR is more desirable.


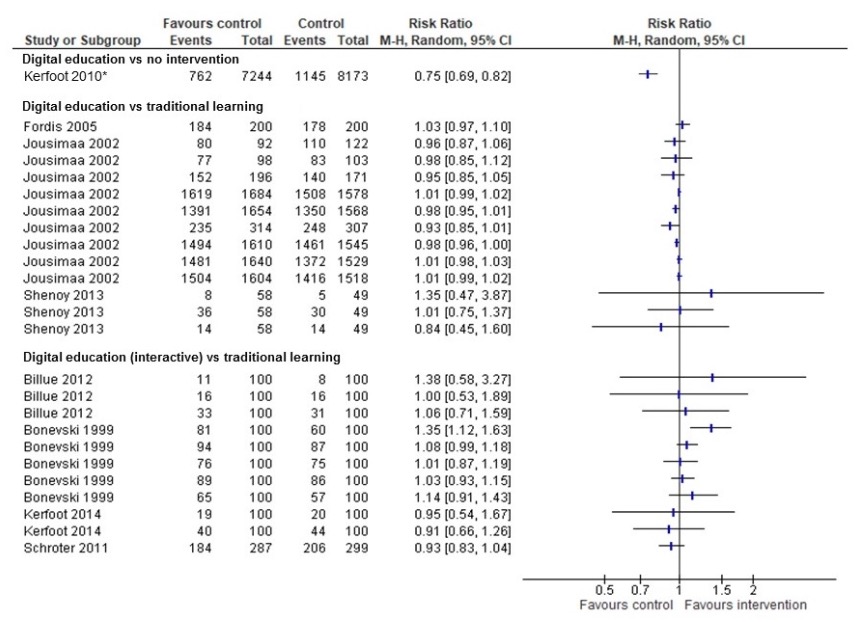

Supplement: Supplementary file 1 — Supplementary information including MEDLINE (Ovid) search strategy, Summary of findings tables and Forest plot of all behavioural change outcomes. (DOCX 155 kb) [file 12916_2019_1370_MOESM1_ESM.docx]
